# The effect of fire ant (Hymenoptera: Formicidae) venom on ecologically relevant bacteria

**DOI:** 10.1093/jee/toaf127

**Published:** 2025-06-19

**Authors:** Ashley Morris, Robert K Vander Meer, Roberto Pereira, Rebecca Baldwin, Satya Chinta

**Affiliations:** Department of Entomology, University of Florida, Gainesville, FL, USA; United States Department of Agriculture, Gainesville, FL, USA; Department of Entomology, University of Florida, Gainesville, FL, USA; Department of Entomology, University of Florida, Gainesville, FL, USA; United States Department of Agriculture, Gainesville, FL, USA

**Keywords:** soybean, bradyrhizobia, alkaloid, nitrogen

## Abstract

Fire ants, *Solenopsis invicta* Buren (Hymenoptera: Formicidae) and *Solenopsis geminata* (Fabricius), have evolved a variety of physiological and chemical defenses against microbe introduction and infection in their colonies. Compounds of most interest are the piperidine alkaloids found in *S. invicta* and *S. geminata*. Alkaloids are produced by the poison gland, stored in the venom sac, and released through the sting. These compounds have antibiotic, antifungal, antiparasitic, antiviral, and hemolytic properties. We hypothesize that fire ants alter the microbiome of their environment through the consistent use of these chemicals in and around their nests, affecting plant–microbe interactions and the rhizobia-legume nodulation process. In this study, *S. invicta* and *S. geminata* worker ant venom extracts were evaluated in disc-diffusion bioassays against the nitrogen-fixing soybean (*Glycine max* L.) (Fabales: Fabaceae) symbionts *Bradyrhizobium japonicum* (Kirchner) (Hyphomicrobiales: Nitrobacteraceae) and *Bradyrhizobium elkanii* Kuykendall and the microbial insecticide *Bacillus thuringiensis* Berliner (Bacillales: Bacillaceae). Venom extracts inhibited the growth of all tested microbes, with *S. geminata* extracts having a greater inhibitory effect than those from *S. invicta*.

## Introduction

Soybeans (*Glycine max* L.) (Fabales: Fabaceae) are a crop of worldwide economic importance and a principal export of the United States, reaching a value of $27.4 billion in 2021 (United States Department of Agriculture [USDA] 2021). Soybeans positively impact soil health and influence the soil microbiome through their symbiotic relationships with certain microbes, such as the aerobic, gram-negative bacteria *Bradyrhizobium* spp. (Pagano and Miransari 2016). During these plant–microbe interactions, the bacteria are enveloped to form a nodule, which provides a carbon-rich environment that sustains the bacteria and stimulates them to fix atmospheric nitrogen into ammonia or ammonium salts (Saranraj et al. 2021). The methods of nodule formation between legumes and rhizobia are summarized by Ferguson (2013).

The two most common modulating bacteria associated with soybeans are *Bradyrhizobium japonicum* (Kirchner) (Hyphomicrobiales: Nitrobacteraceae) and *Bradyrhizobium elkanii* Kuykendall (Jaiswal and Dakora 2019). Although originating in East Asia, they are also considered indigenous to parts of China, Australia, Central America, South America, and the United States (Minamisawa and Mitsui 2000, Sameshima et al. 2003), *Br. japonicum* and *Br. elkanii* are now regarded as cosmopolitan and have been introduced to agricultural ecosystems around the world (Sprent et al. 2017). They are known to increase legume crop yields and are regularly inoculated onto legume seeds or soil (Bogino et al. 2006, Silva et al. 2018). An estimated 15% and 80% of farmers inoculate their soybean seeds in the United States and Brazil, respectively, and *Bradyrhizobium* spp. nodules have been reported across South American, Asian, and African soybean crops (Graham et al. 2004, Saeki 2011, Herrmann et al. 2014, Perticari 2015, Naamala et al. 2016).

Adding nitrogen to the soil via the action of symbiotic rhizobia is preferred since these soil-dwelling microbes already play a dominant role in nitrogen recycling and are the major regulator of inorganic nitrogen levels in soil (Egamberdieva et al. 2018). In contrast, the addition of nitrogen through exogenous crop treatment is known to decrease water quality (Bouraoui et al. 2011), contribute to ozone depletion (United Nations Environment Programme [UNEP] 2017), and pose public health concerns (Hubbard et al. 2004). Minimizing these negative impacts and maintaining soil health is possible through the use of nodulating rhizobia.

The red imported fire ant, *Solenopsis invicta* Buren (Hymenoptera: Formicidae), was shown to interfere with plant-rhizobia symbiosis through disturbance of the soil microbiome when feeding on soybean cotyledons and roots. *Solenopsis invicta* populations in the U.S. range between 100 and 150 colonies per hectare, with over 200,000 workers per colony (Tschinkel 2006), ensuring interactions between fire ants and soybeans from germination to harvest. Soybean plants exposed to *S. invicta* showed less seedling vigor and an 81% reduction in root nodules (Shatters and Vander Meer 2000). This infers a significant interaction between *S. invicta* and the soil microbiome. The reduction in root nodules may be due to the weakening, inhibition, or death of symbiotic microbiota by the antimicrobial alkaloids produced and released.

*Solenopsis invicta* venom is primarily (95%) composed of piperidine alkaloids (Baer et al. 1979), which have been demonstrated to have antibacterial, cytotoxic, hemolytic, insecticidal, antifungal, antiparasitic, and anti-HIV properties (Jouvenaz et al. 1972, Storey et al. 1991, Javors et al. 1993, Howell et al. 2005, Rashid et al. 2013, Khusro et al. 2018, Silva et al. 2020, Wu et al. 2022, Honorato et al. 2024). Examples of antibacterial action by *Solenopsis* spp. produced alkaloids include the suppression of gram-positive *Staphylococcus aureus*, *Streptococcus pneumoniae*, *Stenotrophomonas maltophilia*, and *Enterococcus faecium* growth (Jouvenaz et al. 1972, Sullivan et al. 2009, Yan et al. 2017), as well as the inhibition of quorum signaling in the gram-negative bacterium *Pseudomonas aeruginosa* (Park et al. 2008). Biofilm formation has been found to be inhibited by various piperidine alkaloids in disc-diffusion bioassays (Carvalho et al. 2019). Fire ant venom alkaloids are also present in and around their nest, as fire ants use their venom for direct defense, food procurement, and disease control (Touchard et al. 2016). Behaviors involving venom include inoculation onto eggs during oviposition, aerosol applications onto larvae through gaster-flagging, deposition onto nest material, and direct transmission throughout the colony through trophallaxis (Obin and Vander Meer 1985, Vander Meer and Morel 1995, Chen 2007, Meurville and LeBoeuf 2021, Chen and Du 2022). A review of ant-produced alkaloids and their functions can be found in Fox and Adams (2022).

Here, we evaluate the effect of fire ant worker extracts containing venom alkaloids from invasive *S. invicta*, as well as *Solenopsis geminata* (Fabricius), a species that may be endemic to the southeastern U.S. (Wetterer 2011, Gotzek et al. 2015), on (i) the soybean-associated bacteria, *Br. Japonicum* and *Br. elkanii* and (ii) the bioinsecticide, *Bacillus thuringiensis* Berliner (Bacillales: Bacillaceae) (Bt) through disc-diffusion bioassays. In addition, *Pogonomyrmex badius* (Latreille) (Hymenoptera: Formicidae) extracts were used as a negative control since they are also venom-producing Myrmicine ants, but do not produce alkaloid defensive compounds (Schmidt and Blum 1978, Touchard et al. 2016).

## Materials and Methods

### Fire Ant Colony Collection and Isolation

Monogyne colonies of *S. invicta*, *S. geminata*, and the negative control, *P*. *badius*, were collected in the Gainesville, FL, vicinity between October 2020 and August 2022. For the *Solenopsis* spp., queenright colonies (workers, brood, and queen) were dug from the ground and placed in large buckets whose upper third inner surface was coated with Fluon to prevent ant escape. A dripping apparatus was constructed from a medical intravenous (IV) line and was used to slowly drip water into the buckets. Queen, brood, and workers aggregated on the surface of the water in a raft (Mlot et al. 2011). The raft was removed from the water/bucket with a 15 cm strainer and placed in rectangular plastic trays (36.8 × 50.8 × 12.7 cm) with Fluon coated inner sides. The presence of a queen was verified for each colony, which were then fed a variety of food, including sugar water, tuna in sunflower oil, honey, oranges, grapes, eggs, and German and American roaches (University of Florida Urban Entomology Lab).

Workers from *P. badius* colonies were collected by digging into field colonies and placing the workers and dirt in several trays (36.8 × 50.8 × 12.7 cm) with Fluon coated inner sides. The workers were separated from the dirt by hand, consolidated into a single tray, and fed the same diet as the *Solenopsis* spp. colonies.

### Bacteria Source and Preparation of Bacterial Suspensions

Specific bacteria spore samples were obtained from the USDA Agricultural Research Service Culture Collection as specified here using the Northern Regional Research Laboratory (NRRL) designation: *Bradyrhizobium japonicum* (NRRL B-4507), *Br. elkanii* (NRRL B-4515), and Bt (NRRL B-51075). Cultures were prepared by transferring lyophilized spores into petri dishes containing agar—Yeast Manitol Agar for *Bradyrhizobium* spp. and Potato Dextrose Agar for Bt. The streak plate method was used to isolate genetically uniform colonies. The microbe colonies/Petri dishes were stored in an incubator at 28 °C.

Bacterial suspensions were made by scraping inoculated plates with a scalpel and mixing these bacteria in 10 ml of sterilized water. Suspensions of *Br. japonicum*, *Br. elkanii*, and Bt were made and stored at 5 °C. All suspension densities were estimated with a hemocytometer (Bright-Line—Hausser Scientific, Horsham, PA) and, if necessary, suspensions were diluted or added to, to reach a concentration between 2.0 × 10^6^ and 2.8 × 10^6^ rods per ml.

### Venom Alkaloid Isolation and Preparation for Inhibition Bioassays

One gram of worker ants was randomly collected from their colony trays and weighed in a Fluon-lined container. A mixture of deionized water (1 ml) and hexane (5 ml) was added to a glass container in preparation for worker ant alkaloid extraction (Fox et al. 2013). This general method was validated by Liu et al (2017). The ants (1 g) were vigorously agitated to induce defensive behaviors, e.g., release of venom, prior to being added to the two-phase hexane:water mixture. The solvent mixture containing ants was set aside for 30 min to allow passive extraction. The ants were removed from the mixture with a 10 cm strainer. A glass pipette was used to transfer the hexane (upper) layer into sterile glass vials that were stored at 5 °C. If the hexane evaporated, it was brought back to its original volume (5 ml) before aliquot application onto discs. Venom alkaloids are in the hexane layer and the venom proteins and other water-soluble components are in the water layer. This method was used to obtain venom alkaloids from *S. invicta* and *S. geminata*, and any hexane-soluble components from *P. badius* venom.

### Bacterial Inhibition Bioassays

**Fig. 1.**
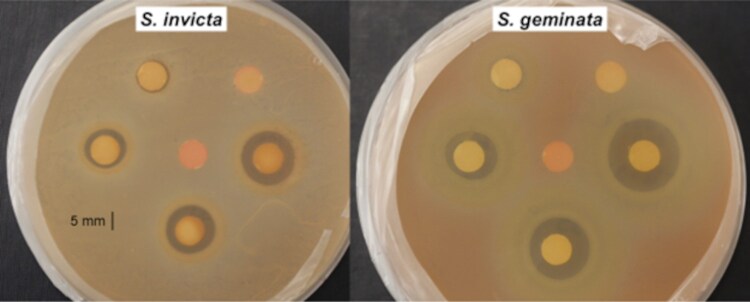
Inhibition of *Br. elkanii* by *S. invicta* (left) and *S. geminata* venom (right) resulting from venom extract disc-diffusion. Each plate contains an untreated control disc (center) surrounded by five treatment discs of increasing concentrations, starting at the top right and moving counterclockwise.

Filter paper discs (7 mm) were sterilized in an autoclave. A micropipette was used to apply the following doses of ant extracts onto separate discs: 1 μl, 3 μl, 10 μl, 33 μl, and 100 μl from the 5 ml/g of ants standard solution. After treatment application, the discs were placed in a fume hood for 1 h to allow the hexane to evaporate. A pipette was used to transfer 250 μl (5 × 10^5^ to 7 × 10^5^ cells/rods) of bacterial suspension into a sterile aerosol sprayer. Then, the suspension was evenly sprayed onto the surface of the agar. This process was repeated with seven plates. Five plates served as replicates and the remaining two were controls. For the treatments, discs of each dose were evenly spaced around the plate as pictured (Fig. 1) with an untreated control disc in the center of the plate. For agar controls, one plate was left untreated to observe the natural bacterial growth pattern. Another plate was used to test for any negative effects of the solvent through the same disc-diffusion procedure with only hexane.

All plates were sealed and stored in an incubator at 26 °C for 24 h before zones of inhibition were measured (mm). The zone of inhibition was defined as the area that had no bacterial growth. Two measurements were recorded for each zone, one in the North/South direction and the other in the East/West direction. The diameter of the disc (7 mm) was subtracted, and the two measurements were averaged to obtain the inhibition data. Experiments were performed with *S. invicta*, *S. geminata*, and *P. badius* extracts to measure their effects on *Br. japonicum*, *Br. elkanii*, and Bt growth.

### Alkaloid Isolation

**Fig. 2.**
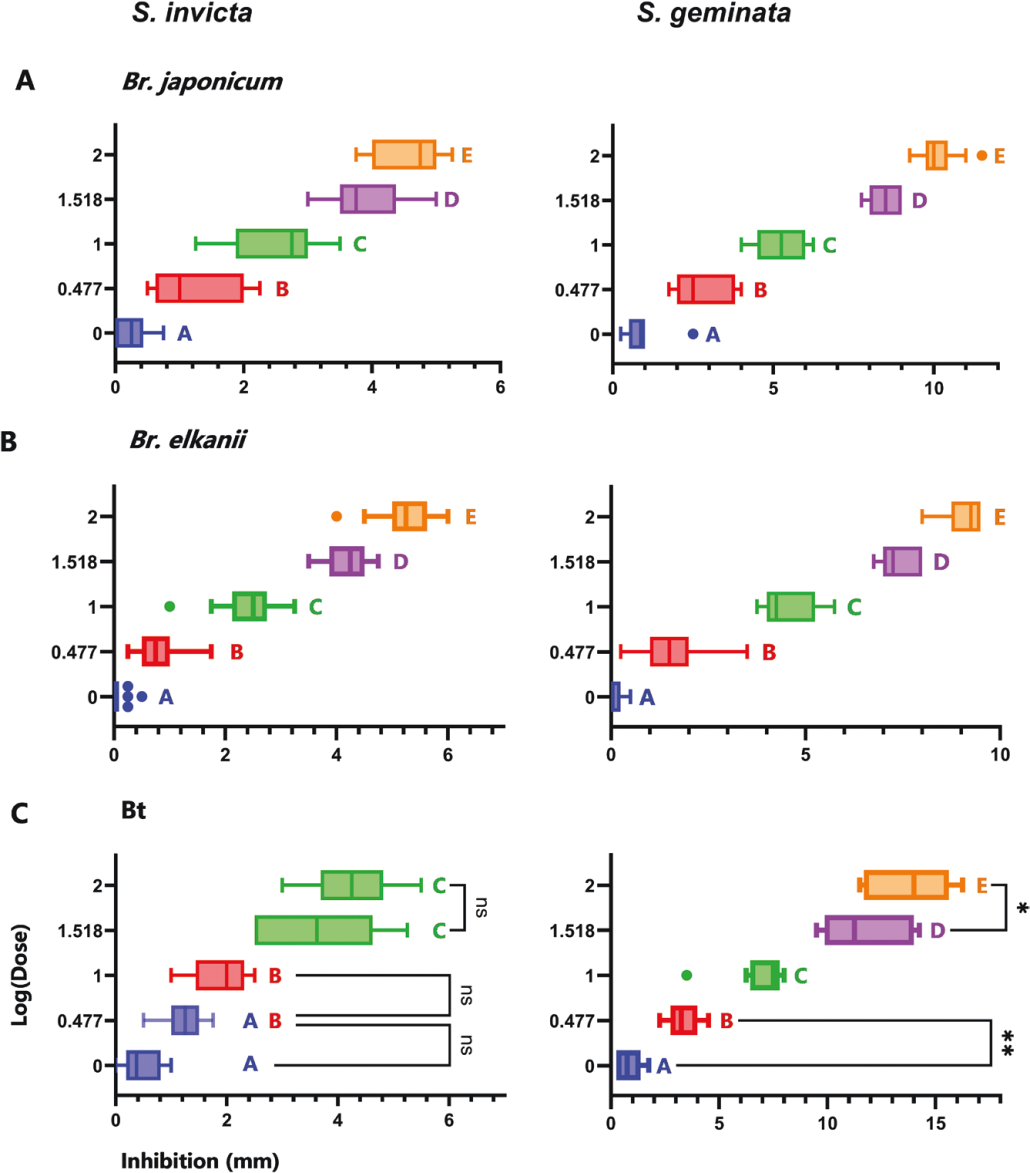
ANOVA and Tukey HSD comparisons between all doses for both extract types against all bacteria. Different letters correspond to significantly differing groups. All doses, unless marked otherwise, significantly differ from one another with *P* < 0.001, showing the dose-dependent effect of the venom. All variance bars represent the standard error of the mean (SEM), **P < 0.01; *P < 0.05; ns = not significant.

The clear differences between the inhibition results for presumably similar amounts of venom alkaloids for *S. invicta* versus *S. geminata* (Fig. 2) drove the need to more precisely isolate and quantitate the alkaloids from the two *Solenopsis* species. This method is a variation of the previously described venom alkaloid isolation and preparation for inhibition bioassays. One gram of ants was placed into a 25-ml beaker containing water (5 ml) and hexane (10 ml). The mixture was gently shaken to ensure all the ants were submerged. After 30 min of soaking, the hexane/water mixture was transferred into a separatory funnel using a pipette (ants were excluded, since the pipette orifice was small). After settling, the bottom aqueous layer was returned to the beaker containing ants and the remaining hexane layer was transferred to a 20 ml scintillation vial (Millipore/Sigma, Burlington, MA). This process was repeated two more times by adding additional hexane (5 ml) into the beaker containing the aqueous layer and ants. The combined hexane layers were evaporated under a stream of nitrogen to a volume of 1 ml. A 2 ng/µl n-tetracosane (Poly Science Corp., Niles, IL) internal standard (IS) stock solution was prepared by adding 0.1 mg tetracosane to 50 ml hexane. A 45 µl aliquot was taken from each sample to be analyzed for alkaloids, then 5 µl of IS (10 ng) was added. The hexane extract with IS was injected onto the GC-MS for analysis. This procedure was repeated to obtain three colony replicates for *S. invicta* and *S. geminata*. All ants in each 1 g replicate were counted after the extraction was complete.

### Alkaloid Quantitation

Gas chromatograph/mass spectral profiles from the *S. invicta* and *S. geminata* extracts were obtained from an Agilent Intuvo 9000 GC system (Santa Clara, CA) equipped with an HP-5 MS ultra-inert nonpolar column, 30 m × 0.25 mm i.d. column, coupled to a 5977 B mass spectral detector and a MassHunter Data Acquisition Workstation version 10.0.368 (Santa Clara, CA). Injector temperature was set at 250 °C. The oven temperature was programmed at 40 °C for 2 min and then to 285 °C at 5 °C/min, followed by a 10 min hold at 285 °C. Venom alkaloids were identified by their retention times, fragmentation patterns, and comparison with synthetic standards, see Vander Meer et al. (2022). An internal standard (n-tetracosane) was used to quantitate the venom components. The area of the alkaloids was determined relative to the area of their associated internal standard, thus giving the relative alkaloid quantity for all samples.

The estimated quantity of alkaloids from *S. invicta* and *S. geminata* worker extracts at the five doses used is shown in  Table 2. Since *S. geminata* extracts were found to contain more ants and, therefore, larger quantities of alkaloid, we recalculated the inhibition per unit mass (μg) of the *S. invicta* extract from our experimental data. From this, we estimated the inhibition of the *S. invicta* venom with the total alkaloid quantity increased to match that of *S. geminata*. The corrected inhibition values are designated as S. invicta* or Si*.

**Table 1.** The zone of bacterial inhibition (mean ± SEM, mm) from the indicated venom extract at five doses against three bacteria is shown. All inhibition values were 100% except as indicated for the 1 μl dose. *Sg* *= S. geminata; Si* *= S. invicta*; *n* = No. of replicates.

A) *Bradyrhizobium japonicum*—Inhibition (mm ± SEM)						
Extract	*n*	1 μl	3 μl	10 μl	33 μl	100 μl
* Sg*	10	0.9250 ± 0.19	2.850 ± 0.29	5.250 ± 0.25	8.450 ± 0.16	10.15 ± 0.20
* Si*	25	0.2500 ± 0.05 ^(65%)^	1.190 ± 0.12	2.470 ± 0.12	3.910 ± 0.13	4.570 ± 0.10
B) *Bradyrhizobium elkanii—*Inhibition (mm ± SEM)						
Extract	***n***	**1 μl**	**3 μl**	**10 μl**	**33 μl**	**100 μl**
* Sg*	15	0.08333 ± 0.040 ^(27%)^	1.517 ± 0.20	4.583 ± 0.18	7.400 ± 0.12	8.981 ± 0.15
* Si*	25	0.05000 ± 0.025 ^(16%)^	0.8300 ± 0.064	2.420 ± 0.10	4.160 ± 0.071	5.240 ± 0.10
C) *Bacillus thuringiensis* Inhibition (mm ± SEM)						
Extract	***n***	**1 μl**	**3 μl**	**10 μl**	**33 μl**	**100 μl**
* Sg*	10	0.8750 ± 0.19	3.350 ± 0.24	6.950 ± 0.43	11.80 ± 0.65	13.80 ± 0.65
* Si*	10	0.4750 ± 0.12 ^(80%)^	1.200 ± 0.11	1.875 ± 0.16	3.650 ± 0.33	4.275 ± 0.24

**Table 2.** The quantity of venom (mean ± SEM, ng) in each extract dose, for *S. invicta* and *S. geminata*, used in disc-diffusion analyses. *Solenopsis geminata* extracts contain more venom by weight at each dose. The difference in the quantity of *S. geminata* and *S. invicta* venom applied to discs increased with increasing doses. *Si* *= S. invicta; Sg* *= S. geminata*; Si* *= *adjusted *S. invicta* venom dose.

Species	1 μl	3 μl	10 μl	33 μl	100 μl
*Sg*	8.884 ± 0.14	26.65 ± 0.41	88.84 ± 1.4	293.2 ± 4.6	888.4 ± 14
*Si*	5.647 ± 0.088	16.94 ± 0.26	56.47 ± 0.88	186.4 ± 2.9	564.7 ± 8.8
Si*	8.884 ± 0.14	26.65 ± 0.41	88.84 ± 1.4	293.2 ± 4.6	888.4 ± 14

### Statistical Analyses

All statistical analyses were performed using GraphPad Prism 10.1.2. For all statistical tests involving bacterial inhibition by dose, a log transformation was applied to the independent variable, dosage, to fulfill the linearity requirement. Linear regression was used to evaluate the linear relationship between dosage and inhibition. For comparisons of the effects of the two extracts on the three bacterial species, a 2-way ANOVA was used to assess the effect of dose and extract type on all bacterial inhibition, with column analyses comparing inhibition by bacterial species and extract type and row analyses comparing inhibition by dosage. Tukey’s HSD was used to determine which bacterial species differed in their inhibition from other bacterial species.

For analyses within bacterial species, a one-sample *t*-test was used to determine which dose caused inhibition significantly different from the mean inhibition from controls, which was zero. An unpaired, 2-sample *t*-test was used to determine statistical significance in inhibition resulting from *S. invicta* and *S. geminata*-produced compounds at each tested dose. A 1-way ANOVA was used to determine statistically significant differences between inhibition resulting from different doses for each bacterium, and Tukey’s honest significance test (HSD) was used to determine which doses resulted in inhibition significantly differing from one another.

For analysis involving alkaloid quantification, an unpaired, two-tailed t-test was used to compare the mean quantity of venom alkaloids extracted from 1 g *S. invicta* and 1 g *S. geminata* workers (*N* = 3). The number of worker ants in 1 g were manually counted for *S. invicta* and for *S. geminata* (*N* = 3). All quantities are reported as mean ± standard error (SEM).

## Results

### Bacterial Susceptibility to *Solenopsis* spp. venom.

The *S. invicta* and *S. geminata* extracts inhibited the growth of the three bacterial species. For all tests, there was a clear zone of inhibition surrounding the treated discs that increased in diameter with increasing dose (Fig. 1). Bacterial growth was consistent across species and replicates, with plates containing very dense bacterial lawns with no gaps apart from the zones of inhibition. The *P. badius* extracts, hexane, and untreated disc controls did not result in bacterial inhibition; therefore, the mean control response was zero. Figure 2 shows all ANOVA results with Tukey’s HSD analyses for *S. invicta* and *S. geminata* extract types against all species of bacteria.

### Comparisons Across Bacterial Species

The susceptibility of the three bacteria to S. *invicta* extracts based on mean inhibition is as follows: *Bradyrhizobium* spp. exhibited a greater area of inhibition than Bt: *Br. japonicum* (2.571 ± 0.15 mm) (*N* = 25) > *Br. elkanii* (2.540 ± 0.18 mm) (*N* = 25) > Bt (2.295 ± 0.23 mm) (*N* = 10). Susceptibility to *S. geminata* extracts differed, with Bt exhibiting the greatest area of inhibition: Bt (7.355 ± 0.73 mm) (*N* = 10) > *Br. japonicum* (5.525 ± 0.50 mm) (*N* = 15) > *Br. elkanii* (4.390 ± 0.40 mm) (*N* = 10). See Table 1 for the mean inhibition (mm) of the three bacteria by both extract types, and at all five doses.

Comparisons of bacterial inhibition by *S. geminata* and *S. invicta* extracts against the three bacterial species revealed that S*. geminata* extracts resulted in greater variation in their inhibition. The two-way ANOVA demonstrated a statistically significant interaction between the effects of dose (*F* = 12.20; df = 124, 338; *P* < 0.001) and extract type and bacterial species (*F* = 104.5; df = 5, 338; *P* < 0.001) on inhibition. Tukey’s HSD test for multiple comparisons showed a significant difference in inhibition from *S. geminata* extracts between all bacteria: *Br. japonicum* and *Br. elkanii* (*P* = 0.004), *Br. Japonicum* and Bt (*P* < 0.001), *Br. elkanii* and Bt (*P* < 0.001). There were no significant differences in inhibition resulting from *S. invicta* venom for all bacterial comparisons.

### Venom Quantification

**Fig. 3.**
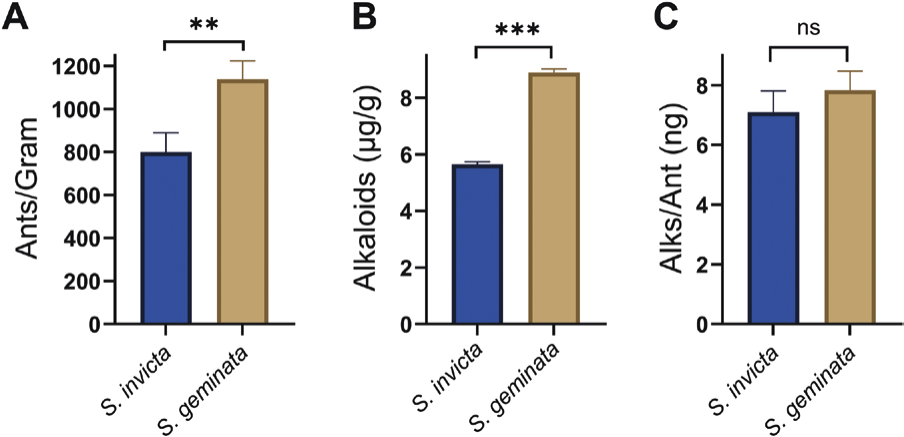
Quantity (mean ± SEM) of ants or venom produced by *S. invicta* and *S. geminata*. (A) The number of workers in one gram of *S. invicta* are compared to the number of workers in one gram of *S. geminata*. (B) The quantity of alkaloids is shown for one gram of ants (μg/ant) from *S. invicta and S. geminata*, and (C) The quantity of alkaloids (ng/ant) is shown for S*. invicta* and *S. geminata*. ***P < 0.001; **P < 0.01; ns = not significant.

The number of worker ants per gram was significantly greater for *S. geminata* (1139 ± 49) (*N* = 3) than *S. invicta* (801.0 ± 52) (*N* = 3) (t = 4.733; df = 4; *P* = 0.009) (Fig. 3A). *Solenopsis geminata* had 42% more workers in a gram than *S. invicta*. Similarly, the micrograms of alkaloids in one gram of worker ants were higher for *S. geminata* (8.884 ± 0.14 μg) than *S. invicta* (5.647 ± 0.088 μg), averaging 36% more alkaloid per gram of ants, (Fig. 3B). The unpaired, 2-tailed *t*-test confirmed that these venom alkaloid quantities significantly differed from one another (*t* = 19.00; df = 4, *P* < 0.001).

**Fig. 4.**
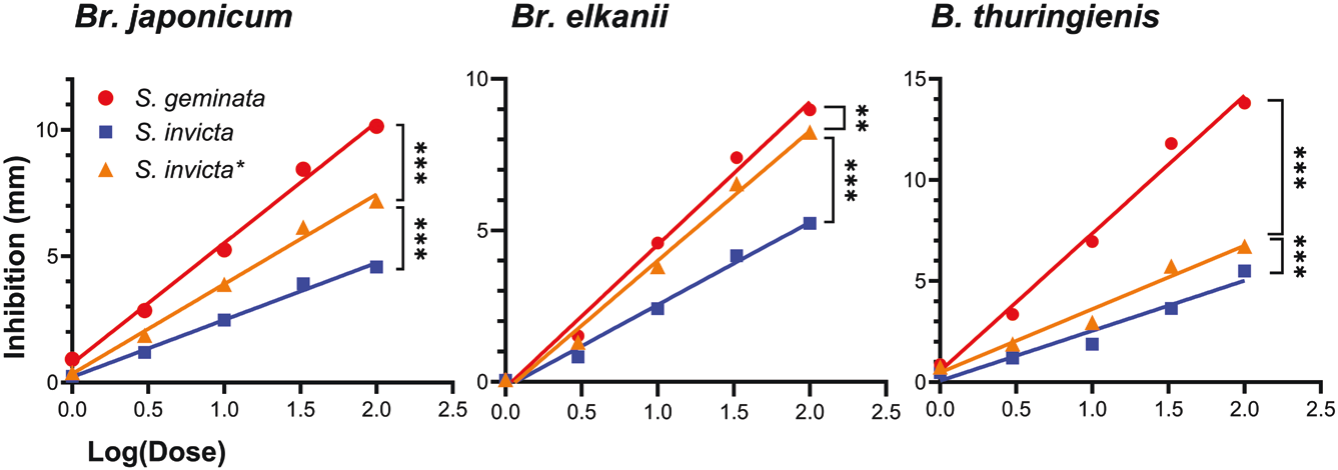
Linear regression models are shown for extract dose vs. inhibition for each bacterium. *Solenopsis geminata* venom resulted in a larger zone of inhibition for the three bacteria, significantly differing from both *S. invicta* and corrected S. invicta* inhibition values. ***P < 0.001; **P < 0.01.

When the quantity of alkaloids per ant was calculated, however, the differences between the two *Solenopsis* species disappeared (*t* = 1.322; df = 4; *P* = 0.257) (Fig. 3C). The larger quantity of alkaloids extracted from one gram of *S. geminata* was due to worker numbers and not due to *S. geminata* workers synthesizing a greater quantity of alkaloids. The net result is that the *S. geminata* extract had a higher concentration of alkaloids and therefore, greater bacterial inhibition than the *S. invicta* extract. We hypothesized that the alkaloids from *S. invicta* and *S. geminata* have equivalent antimicrobial activity and calculated the inhibition per unit mass of the *S. invicta* extract from our actual data. From this, we estimated the inhibition of the *S. invicta* venom with the concentration increased to match that of *S. geminata*. The results are designated as S. invicta* or Si* and are shown in Fig. 4 and Table 2.

### Comparisons Within Bacterial Species

#### Bradyrhizobium japonicum

The mean value of inhibition at the lowest dose (1 μl) was significantly higher than zero for both extracts: *S. invicta* (*N* = 25) (*t* = 4.595; df = 19; *P* < 0.001), and *S. geminata* (*N* = 10) (*t* = 4.772; df = 9; *P* < 0.001). The 1-way ANOVA revealed a statistically significant difference in inhibition between at least two doses for *S. invicta* (*F* = 243.3; df = 4, 115; *P* < 0.001) and *S. geminata* (*F* = 288.1; df = 4, 45; *P* < 0.001). Tukey’s HSD Test for multiple comparisons found that the mean value of inhibition was significantly different between all doses for all extract types (*P* < 0.001, Fig. 2A).

Inhibition resulting from *S. geminata* extracts was greater than that of *S. invicta* for all doses (Fig. 4). The unpaired, two-tailed t-test revealed that, even at the 1 μl dose, there was a significant difference in mean inhibition resulting from *S. geminata* and *S. invicta* (*t* = 4.344; df = 28; *P* < 0.001). The difference in mean inhibition increased with increasing dose, with *P* < 0.001 for all *t*-test comparisons to *S. geminata* at the higher doses (3, 10, 33, and 100 μl) (For all *t*-test results, see Supplementary Table 1).

#### Bradyrhizobium elkanii

The mean value of *Br. elkanii* inhibition by at the lowest tested dose (1 μl) was not significantly higher than zero for *S. invicta* (*N* = 25) (*t* = 2.000; df = 24; *P* = 0.057) nor *S. geminata* (*N* = 15) (*t* = 2.092; df = 14; *P* = 0.055). The 3 μl dose, however, significantly differed from zero for all comparisons: *S. invicta* (*t* = 12.95; df = 24; *P* < 0.001), *S. geminata* (*t* = 7.625; df = 14; *P* < 0.001). The 1-way ANOVA revealed that there was a statistically significant difference in inhibition between at least two doses for *S. invicta* (*F* = 773.5; df = 4, 120; *P* < 0.001) and *S. geminata* (*F* = 627.6; df = 4, 68; *P* < 0.001). Tukey’s HSD Test for multiple comparisons found that the mean value of inhibition was significantly different between all doses for all extract types, with *P* < 0.001 for all dose comparisons (Fig. 2B).

Inhibition by *S. geminata* extracts was stronger than that of *S. invicta* for all doses higher than 1 μl (Fig. 4). At the 1 μl dose, there was no significant difference in mean inhibition between *S. geminata* and *S. invicta* extracts (*t* = 0.7475; df = 38; *P* = 0.4593). A comparison between mean inhibition by species at the 3 μl dose, however, displayed a significant difference (*t* = 3.949; df = 38; *P* < 0.001). The difference in mean inhibition increased with increasing dose, with *P* < 0.001 for all *t*-test comparisons to *S. geminata* for the higher doses.

#### Bacillus thuringiensis

The mean value of inhibition by at the lowest tested dose (1 μl) was significantly higher than zero for *S. invicta* (*N* = 10) (*t* = 3.943; df = 9; *P* = 0.003) and *S. geminata* (*N* = 10) (*t* = 4.583; df = 9; *P* = 0.001). The 1-way ANOVA revealed that there was a statistically significant difference in inhibition between at least two doses for *S. invicta* (*F* = 59.44; df = 4, 45; *P* < 0.001) and *S. geminata* (*F* = 133.1; df = 4, 45; *P* < 0.001). Tukey’s HSD Test for multiple comparisons found that the inhibition from *S. invicta* resulted in three significantly different groups, with the lowest three doses (*P* = 0.122; *P* = 0.171) and two highest doses (*P* = 0.234) demonstrating no significant difference from one another (Fig. 2C). Inhibition from Si* resulted in three significantly different groups, with the two lowest doses (*P* = 0.1219), the 10 μl and 33 μl doses (*P* = 0.1713), and the two highest doses (*P* = 0.2344) not significantly differing from one another. All other dose comparisons within extract types resulted in significantly differing groups (*P* < 0.001).

Inhibition by *S. geminata* extracts was stronger than that of *S. invicta* for all doses higher than 1 μl (Fig. 4). At the 1 μl dose, there was no significant difference in inhibition between *S. geminata* and *S. invicta* (*t* = 1.772; df = 18; *P* = 0.0934). A comparison between mean inhibition by species at the 3 μL dose, however, displayed a significant difference: *S. geminata* and *S. invicta* (*t* = 8.242; df = 18; *P* < 0.001). The difference in mean inhibition increased with increasing dose, with *P* < 0.001 for all *t*-test comparisons to *S. geminata* for the higher doses. The inhibition resulting from the 100 μl dose had the highest difference in mean inhibition by species than any other bacterium.

### Linear Regression Analyses and Comparisons with Adjusted Si* Values

The Si* results for *S. invicta* were significantly increased from the *S. invicta* experimental results: *Br. japonicum* (*P* < 0.001), *Br. elkanii* (*P* < 0.001), and Bt (*P* < 0.001). However, the Si* results were also significantly lower than the inhibition results for *S. geminata*: *Br. japonicum* (*P* < 0.001), *Br. elkanii* (*P* = 0.002), and Bt (*P* < 0.001). Figure 4 shows linear models of the inhibition resulting from *S. geminata*, *S. invicta*, and Si*.

For *Br. japonicum*, the fitted regression model for *S. invicta* experimental results was Y = 2.259*X + 0.2202. The overall regression results were statistically significant (*R*^2^ = 0.886; *F*(1, 118) = 919.5; *P* < 0.001), showing that dose significantly predicted inhibition. The overall regression for Si* had a slightly increased slope (Y = 3.553X + 0.3464) (*R*^2^ = 0.886; *F*(1, 118) = 919.5; *P* < 0.001). The fitted regression model for *S. geminata* results was Y = 4.775X + 0.7537. The overall regression results were also statistically significant (*R*^2^ = 0.956; *F*(1, 48) = 1054; *P* < 0.001). Inhibition resulting from *S. geminata* extracts was greater than that of Si* for all doses. The unpaired, 2-tailed *t*-test revealed that, at the 1 μl dose, there was a significant difference in mean inhibition resulting from *S. geminata* and Si* (*t* = 2.926; df = 28; *P* = 0.0067). The difference in mean inhibition increased with increasing dose, with *P* < 0.001 for all *t*-test comparisons to *S. geminata* at the higher doses (3, 10, 33, and 100 μl).

For *Br. elkanii*, the fitted regression model for *S. invicta* experimental results was Y = 2.724X - 0.1812. The overall regression results were statistically significant (*R*^2^ = 0.953; *F*(1, 123) = 2485; *P* < 0.001), showing that dose significantly predicted inhibition. The overall regression for Si* had a significantly increased slope (Y = 4.285X - 0.2851) (*R*^2^ = 0.953; *F*(1, 123) = 2485; *P* < 0.001). The fitted regression model for *S. geminata* results was Y = 4.719X - 0.1947. The overall regression results were statistically significant (*R*^2^ = 0.963; *F*(1, 71) = 1825; *P* < 0.001). An unpaired, 2-tailed *t*-test revealed that inhibition resulting from *S. geminata* extracts were only significantly greater than that of Si* for at the 10 μl (*t* = 3.108; df = 38; *P* = 0.0036) and 33 μl (*t* = 4.985; df = 38; *P* < 0.001) doses.

For *B. thuringiensis*, the fitted regression model for *S. invicta* experimental results was Y = 1.996X + 0.3004. The overall regression results were statistically significant (*R*^2^ = 0.816; *F*(1, 48) = 212.4; *P* < 0.001), showing that dose significantly predicted inhibition. The overall regression for Si* had a significantly increased slope (Y = 3.141X + 0.4726) (*R*^2^ = 0.816; *F*(1, 48) = 212.4; *P* < 0.001). The fitted regression model for *S. geminata* results was Y = 6.814X + 0.5466. The overall regression results were also statistically significant (*R*^2^ = 0.911; *F*(1, 48) = 491.2; *P* < 0.001). The unpaired, 2-tailed *t*-test revealed that inhibition resulting from *S. geminata* extracts were significantly greater than that of Si* at the 3 μl dose (*t* = 4.983; df = 18; *P* < 0.001). The difference in mean inhibition increased with increasing dose, with *P* > 0.001 for all *t*-test comparisons to *S. geminata* for the higher doses.

## Discussion

The effectiveness of 2-methyl-6-alkyl and alkenyl piperidine alkaloids as antibiotic agents was established early in *S. invicta*’s invasion of the southern United States and suggests that these alkaloids are responsible for the observed antibacterial activity from *S. invicta* extracts (Blum et al. 1958, Jouvenaz et al. 1972, Sullivan et al. 2009, Carvalho et al. 2019). The inhibition of gram-negative *Bradyrhizobium* spp. and gram-positive Bt growth by these extracts shows that the alkaloids have broad spectrum activity against soil-dwelling bacteria, as these two bacteria differ in their biology but are both found naturally in soils throughout the world (Lambert and Peferoen 1992, Sprent et al. 2017). This also explains specific observations of reduced nodule formation in soybeans exposed to *S. invicta* colonies (Shatters and Vander Meer 2000).

The use of venom alkaloids for food procurement and nest hygiene through venom aerosol formation via gaster vibration and other forms of venom release (Obin and Vander Meer 1985) suggest that these compounds are prevalent in and around fire ant colonies, where they alter the soil microbiome. The low quantity of venom needed to achieve bacterial inhibition is notable. *Bradyrhizobium japonicum* and Bt exhibited inhibition at the lowest dose containing about 6 ng venom. This represents approximately 1% of the quantity released from a single *S. invicta* sting (560 ng) and 0.0003% of the venom reserves of a single worker (18.1 μg) (Haight and Tschinkel 2003). Given an estimated 200,000 ants per colony and 60 colonies per acre (Tschinkel 2006), each harboring about 576 mg at any given point in time, the cumulative venom biomass per hectare is approximately 85.4 g.

Before the adjusted Si* values were calculated, *S*. *geminata* extracts produced greater inhibition than *S. invicta* extracts for all bacteria. Since there were more ants present in one gram of *S. geminata* there was a higher concentration of alkaloids in the *S. geminata* extract compared to the *S. invicta* extract, resulting in increased inhibition. The corrected values equalizing the alkaloid concentrations (Si*) did proportionally increase inhibition; however, the *S. geminata* venom zones of inhibition for *Br. japonicum* and Bt were still significantly greater. Thus, showing that *S. geminata* alkaloid extracts have a greater negative effect on bacterial inhibition than equivalent amounts of *S. invicta* alkaloid extracts. *Bradyrhizobium elkanii*, however, was inhibited similarly by both extract types after Si* adjustments.

Comparisons between extracts are indicative that fire ant species differ qualitatively in their venom alkaloid profiles, which likely influence their effects against bacteria. The variation in inhibition could result from their innate structure and evolutionary history with fire ants and the soil used for nesting. The higher susceptibility of *Br. japonicum*, suggests that soybean fields predominantly inoculated with *Br. japonicum*, including in the United States and Asia, may experience greater decline in plant vigor due to fire ant infestations compared to areas utilizing *Br. elkanii*. The former is considered the more efficient and is a widely applied soybean symbiont (Hungria and Bohrer 2000). Countries where *S. geminata* is an established invasive species are likely to observe more rhizobia-legume symbiosis disruption. As we demonstrate here, fire ant infestations not only affect soybean plants through direct feeding but also through soil microbiome modification in and around nests, resulting in a deficiency of nitrogen-fixing microbes and plant-soluble nitrogen.

*Solenopsis* spp. differ in the components of their venom. The alkaloid composition of *S*. *invicta* has been known for decades due to its importance as an invasive species throughout the southeastern U.S., as well as its aggressive behavior and potent sting. The four major *S. invicta* worker alkaloids are *trans*-piperidines: 2-methyl-6-tridecyl-piperidine, 2-methyl-6-tridecenyl-piperidine, 2-methyl-6-pentadecyl-piperidine, and 2-methyl-6-pentadecenyl-piperidine. In addition, there are minor amounts of 2-methyl-6-undecyl-piperidine and 2-methyl-6-heptadecenyl-piperidine. *Solenopsis geminata* has long been reported to produce *cis*- and *trans*-2-methyl-6-undecyl-piperidines (Brand et al. 1973), which makes *S. geminata* alkaloids almost completely unique from those produced by *S. invicta*. The *trans*-undecyl-piperidine consistently showed greater bacterial inhibition than piperidines with longer chain lengths—as produced by *S. invicta* (in Xu and Chen 2023). This by itself could explain our results showing greater gram-positive bactericidal activity with *S. geminata* extracts versus those from *S. invicta*. The *S. geminata* alkaloid possibilities were recently expanded to the following pyridines: 6-undecyl-pyridine, 2-methyl-6-undecyl-pyridine, and 2-methyl-6-(1)-undecenyl-pyridine, plus 2-methyl-6-undecyl-Δ^1,2^-piperideine (Vander Meer et al. 2022).

The pyridine ring structure occurs in many natural products, such as nicotine, niacin, and pyridoxine, and both synthetic and natural pyridines have been utilized for industrial, agricultural, and medical applications due to their protein-binding capacity (De et al 2022). Pyridines have also been widely reported as having antibacterial, antiviral, and antitumor properties (Marinescu and Popa 2022). The broad-spectrum biocidal activity of these compounds likely further contributes to heightened bacterial inhibition in the presence of *S. geminata* alkaloids. Future experiments include bacterial inhibition experiments with (i) isolated alkaloids from each of the two *Solenopsis* species and (ii) synthetic *S. invicta* and *S. geminata* alkaloids.

## Supplementary material

Supplementary material is available at *Journal of Economic Entomology* online.

toaf127_Supplementary_Tables_1
