# Supplementary material for: The effect of fire ant (Hymenoptera: Formicidae) venom on ecologically relevant bacteria
Source: J Econ Entomol. 2025 Jun 19;118(5):2123–32. doi: 10.1093/jee/toaf127 (PMC12602152; doi:10.1093/jee/toaf127)
Supplement: toaf127_Supplementary_Tables_1 [file toaf127_supplementary_tables_1.docx]

**Supplementary Table 1**: Results of t-tests between inhibition (mm) by *S. geminata* and *S. invicta* for each bacterium at every dose. *Si** *= S. invicta* corrected

| Bacterium | Comparison | Dose | M1 | M2 | t | df | P | Significance |
| --- | --- | --- | --- | --- | --- | --- | --- | --- |
| *Br. japonicum* | *S. invicta* | 1 | 0.25 | 0.925 | 4.344 | 28 | < 0.001 | s |
| *Br. japonicum* | *S. invicta* | 3 | 1.19 | 2.85 | 6.216 | 33 | < 0.001 | s |
| *Br. japonicum* | *S. invicta* | 10 | 2.47 | 5.25 | 11.05 | 33 | < 0.001 | s |
| *Br. japonicum* | *S. invicta* | 33 | 3.91 | 8.45 | 19.85 | 33 | < 0.001 | s |
| *Br. japonicum* | *S. invicta* | 100 | 4.57 | 10.15 | 27.49 | 33 | < 0.001 | s |
| *Br. japonicum* | *Si** | 1 | 0.3933 | 0.925 | 2.926 | 28 | 0.0067 | s |
| *Br. japonicum* | *Si** | 3 | 1.872 | 2.85 | 2.726 | 33 | 0.0102 | s |
| *Br. japonicum* | *Si** | 10 | 3.886 | 5.25 | 3.928 | 33 | < 0.001 | s |
| *Br. japonicum* | *Si** | 33 | 6.151 | 8.45 | 6.761 | 33 | < 0.001 | s |
| *Br. japonicum* | *Si** | 100 | 7.19 | 10.15 | 10.57 | 33 | < 0.001 | s |
| *Br. elkanii* | *S. invicta* | 1 | 0.05 | 0.08333 | 0.7475 | 38 | 0.4593 | ns |
| *Br. elkanii* | *S. invicta* | 3 | 0.83 | 1.517 | 3.949 | 38 | < 0.001 | s |
| *Br. elkanii* | *S. invicta* | 10 | 2.42 | 4.453 | 11.3 | 38 | < 0.001 | s |
| *Br. elkanii* | *S. invicta* | 33 | 4.16 | 7.4 | 24.79 | 38 | < 0.001 | s |
| *Br. elkanii* | *S. invicta* | 100 | 5.24 | 8.981 | 20.9 | 36 | < 0.001 | s |
| *Br. elkanii* | *Si** | 1 | 0.07866 | 0.08333 | 0.07852 | 38 | 0.9378 | ns |
| *Br. elkanii* | *Si** | 3 | 1.306 | 1.517 | 1.049 | 38 | 0.301 | ns |
| *Br. elkanii* | *Si** | 10 | 3.807 | 4.583 | 3.108 | 38 | 0.0036 | s |
| *Br. elkanii* | *Si** | 33 | 6.545 | 7.4 | 4.985 | 38 | < 0.001 | s |
| *Br. elkanii* | *Si** | 100 | 8.244 | 8.981 | 2.939 | 36 | 0.0057 | ns |
| Bt | *S. invicta* | 1 | 0.475 | 0.875 | 1.772 | 18 | 0.0934 | ns |
| Bt | *S. invicta* | 3 | 1.2 | 3.35 | 8.242 | 18 | < 0.001 | s |
| Bt | S. invicta | 10 | 1.875 | 6.95 | 11.15 | 18 | < 0.001 | s |
| Bt | S. invicta | 33 | 3.65 | 11.8 | 11.12 | 18 | < 0.001 | s |
| Bt | S. invicta | 100 | 4.275 | 13.8 | 13.78 | 18 | < 0.001 | s |
| Bt | *Si** | 1 | 0.7473 | 0.875 | 0.4747 | 18 | 0.6407 | ns |
| Bt | *Si** | 3 | 1.888 | 3.35 | 4.983 | 18 | < 0.001 | s |
| Bt | *Si** | 10 | 2.95 | 6.95 | 8.092 | 18 | < 0.001 | s |
| Bt | *Si** | 33 | 5.742 | 11.8 | 7.242 | 18 | < 0.001 | s |
| Bt | *Si** | 100 | 6.726 | 13.8 | 9.428 | 18 | < 0.001 | s |
